# Esophageal Cancer Development: Crucial Clues Arising from the Extracellular Matrix

**DOI:** 10.3390/cells9020455

**Published:** 2020-02-17

**Authors:** Antonio Palumbo, Nathalia Meireles Da Costa, Bruno Pontes, Felipe Leite de Oliveira, Matheus Lohan Codeço, Luis Felipe Ribeiro Pinto, Luiz Eurico Nasciutti

**Affiliations:** 1 Laboratório de Interações Celulares, Instituto de Ciências Biomédicas, Universidade Federal do Rio de Janeiro Prédio de Ciências da Saúde—Cidade Universitária, Ilha do Fundão, Av. Carlos Chagas, 373—bloco F, sala 26, Rio de Janeiro CEP: 21941-902, RJ, Brazil; matheuslohan@hotmail.com (M.L.C.); luiz.nasciutti@histo.ufrj.br (L.E.N.); 2 Programa de Carcinogênese Molecular, Instituto Nacional de Câncer—INCA, Rua André Cavalcanti, 37—Centro, Rio de Janeiro CEP: 20231-050, RJ, Brazillfrpinto@inca.gov.br (L.F.R.P.); 3 Laboratório de Pinças Óticas (LPO-COPEA), Instituto de Ciências Biomédicas, Universidade Federal do Rio de Janeiro, Rio de Janeiro 21941-902, RJ, Brazil; brunoaccpontes@gmail.com; 4 Laboratório de Proliferação e Diferenciação Celular, Instituto de Ciências Biomédicas (ICB), Universidade Federal do Rio de Janeiro, Rio de Janeiro 21941-902, RJ, Brazil; felipe@histo.ufrj.br

**Keywords:** esophageal carcinogenesis, extracellular matrix, stiffness, remodeling, adhesion, metalloproteinases, glycoproteins, proteoglycans

## Abstract

In the last years, the extracellular matrix (ECM) has been reported as playing a relevant role in esophageal cancer (EC) development, with this compartment being related to several aspects of EC genesis and progression. This sounds very interesting due to the complexity of this highly incident and lethal tumor, which takes the sixth position in mortality among all tumor types worldwide. The well-established increase in ECM stiffness, which is able to trigger mechanotransduction signaling, is capable of regulating several malignant behaviors by converting alteration in ECM mechanics into cytoplasmatic biochemical signals. In this sense, it has been shown that some molecules play a key role in these events, particularly the different collagen isoforms, as well as enzymes related to its turnover, such as lysyl oxidase (LOX) and matrix metalloproteinases (MMPs). In fact, MMPs are not only involved in ECM stiffness, but also in other events related to ECM homeostasis, which includes ECM remodeling. Therefore, the crucial role of distinct MMPs isoform has already been reported, especially MMP-2, -3, -7, and -9, along EC development, thus strongly associating these proteins with the control of important cellular events during tumor progression, particularly in the process of invasion during metastasis establishment. In addition, by distinct mechanisms, a vast diversity of glycoproteins and proteoglycans, such as laminin, fibronectin, tenascin C, galectin, dermatan sulfate, and hyaluronic acid exert remarkable effects in esophageal malignant cells due to the activation of oncogenic signaling pathways mainly involved in cytoskeleton alterations during adhesion and migration processes. Finally, the wide spectrum of interactions potentially mediated by ECM may represent a singular intervention scenario in esophageal carcinogenesis natural history and, due to the scarce knowledge on the cellular and molecular mechanisms involved in EC development, the growing body of evidence on ECM’s role along esophageal carcinogenesis might provide a solid base to improve its management in the future.

## 1. Introduction

**Figure 1:**
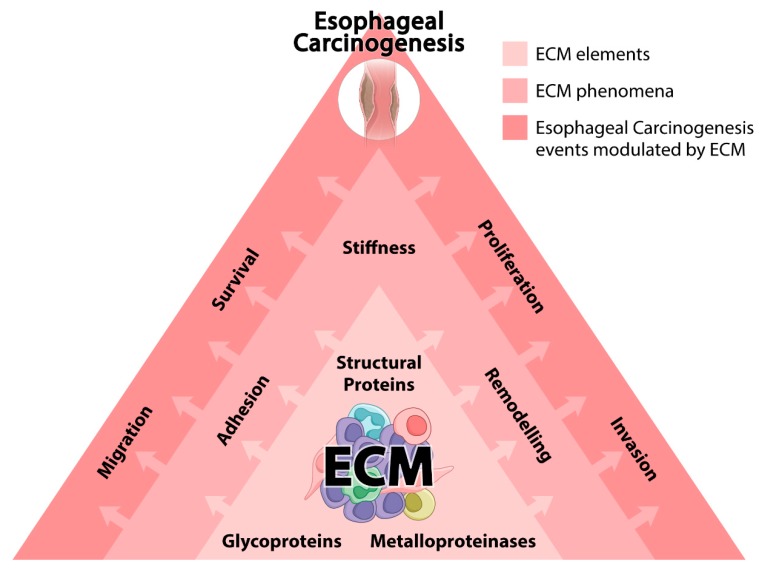
Extracellular matrix (ECM) and esophageal carcinogenesis. The figure schematically represents how ECM may affect the genesis and/or development of esophageal tumors. The first layer represents the main classes of ECM constituting proteins involved in esophageal carcinogenesis. The second layer represents ECM phenomena modulated in esophageal tissues as a consequence of the altered protein constitution and function of this compartment. The third layer represents the crucial cellular events impacted by structural and functional alterations occurring in ECM, which may play a crucial role in esophageal carcinogenesis. Arrows indicate the dynamism and hierarchy between the three layers of events.

The high level of organization among multicellular organisms is largely attributed to a constant process of communication between different cellular types [1,2]. In fact, due to this highly organized communication system, a myriad of chemical signals is stratified, thus integrating physically distant compartments in a functional organism [3,4]. However, the physical and chemical integration is not only based on the establishment of cell–cell communications, but also on the communication between cells and their surrounding microenvironment, particularly the extracellular matrix (ECM) [5,6]. ECM can be defined as a complex non-cellular compartment, present within tissues and organs, providing physical scaffolding, as well as biochemical and biomechanical signals required for several biological processes [1]. In this sense, the establishment of interactions between cells and ECM is crucial for morphogenesis and homeostasis maintenance, besides presenting an important role in the genesis and progression of distinct diseases, including cancer [7,8]. Thus, the relevance of ECM in cancer has strongly increased over the last years, and it is now well demonstrated that it does not represent only a mere physical compartment, but a functional protein network, capable of modulating the fate of key events during carcinogenesis, such as cell survival, apoptosis, proliferation, angiogenesis, migration, and invasion, among others [7,8,9,10,11,12]. Additionally, several biological aspects of ECM (adhesion, stiffness, remodeling, growth factor release, and others) may cooperate and stimulate the malignant behavior of cancer cells by a feedback mechanism that is established between tumors and their surrounding microenvironment [13,14]. Moreover, in the last years, it has been reported that ECM plays a relevant role in esophageal cancer (EC) development, being this compartment related to several aspects of its genesis and progression [15]. EC is a highly incident and lethal cancer, taking the sixth position in mortality in men, among all tumor types worldwide [16]. This lethal tumor confers a 5-year survival rate of about 15–25% of patients, demonstrating its poor prognosis [17]. There are two main EC histopathological subtypes: esophageal squamous cell carcinoma (ESCC) and esophageal adenocarcinoma (EAC), which widely differ considering populations affected, etiological factors, and molecular alterations, among others. Although ESCC development is highly associated with tobacco and alcohol abuse and ingestion of high temperature beverages, for EAC, the main associated risk factors are obesity, gastroesophageal reflux disease (GERD), and Barrett’s esophagus (BE), an intestinal metaplasia where the normal stratified squamous esophageal epithelium is replaced by a columnar intestinal-like one [18,19]. ESCC represents the predominant EC histotype; nevertheless, along with the increase in obesity rates in some western countries, the incidence of EAC has increased sharply over the past few decades [20,21]. Although EC is a remarkably incident and lethal cancer, the knowledge on its biology is still scarce. EC poor prognosis is directly associated with its late detection, which is a consequence of the lack of clinical symptoms in early tumor stages. Thus, the deeper understanding of the mechanisms involved in its genesis and/or progression may be useful in identifying potential markers for diagnosis and prognosis, as well as potential therapeutic targets. Therefore, the reciprocal interactions that occur between the cells and the surrounding ECM orchestrate a complex cascade of events during esophageal malignant transformation, where the adhesion mediated by glycoproteins and proteoglycans triggers signaling pathways, which induce the expression and activation of catalytic enzymes that, in turn, promote structural alterations in ECM stiffness and remodeling [15]. These alterations culminate in the activation of signaling pathways in a feedback loop mechanism (Figure 1). In this context, the interactions between EC and ECM may shed some light on the cellular and molecular mechanisms that govern the malignant development of these tumors. Thus, the aim of this review was to compile existing data of crucial phenomena related to ECM, focusing specifically on the stiffness, remodeling, and adhesion/migration events under the context of esophageal carcinogenesis, as presented in the following sections.

## 2. EC and ECM

### 2.1. Structural Proteins and ECM Stiffness

The tumor microenvironment plays a key role in tumor development and progression [15,22,23,24,25,26]. This dynamic environment is formed by several cellular components that include not only cancer cells, but also fibroblasts, and immune and endothelial cells [15]. Moreover, one of the most important constituents of the tumor microenvironment is the ECM, a meshwork of proteins and glycosaminoglycans surrounding tumor cells [12]. ECM provides the biochemical and mechanical support for tumor progression [12,27,28]. The expression of several ECM proteins has been shown to be upregulated in tumors, of which, the most abundant is type I collagen [29]. This structural protein not only fills up the interstitial spaces between cancer cells, but it also changes the mechanical properties of cancer tissues, particularly by increasing their stiffness and providing tissues with tensile strength and resistance to deformation [12,30]. The increase in tissue stiffness occurs not only due to the increased deposition and cross-linking of thick fibers of type I collagen together with other proteins, such as fibronectin and types III and IV collagens, but also by the increase in cross-linking capacity of these meshworks [29]. These modifications involve several post-translational changes in the molecule that take place inside the cells, followed by reactions that occur in the ECM [31]. For instance, lysine or hydroxylysine residues in collagen molecule can undergo oxidative deamination catalyzed by lysyl oxidase (LOX) enzymes at the ECM, leading to the formation of covalent intermolecular cross-links between neighboring collagen molecules [31]. Although type I collagen was traditionally considered as a physical barrier to prevent tumor cell dissemination, mainly because early studies in the field showed that collagen molecules should be degraded to allow tumor progression, it is now evident that type I collagen is actively involved in tumor invasion [32]. It is well-established that the increase in ECM structure and stiffness is directly associated with tumor progression. The so-called “tumor stiffening” is due to the deposition and cross-linking of several ECM molecules [29,33,34,35,36,37]. Huge amounts of ECM molecules are deposited during EC development [38]. Thus, the constituents of the ECM can have a lasting effect on cancer cells. Indeed, ECM remodeling creates a reorganized environment that promotes tumor progression by destabilizing cell polarity and cell–cell adhesions and by increasing growth factor signaling [32], which impact on tumor gene expression, differentiation, proliferation, migration, and responses to treatments [32]. Type I collagen protein secretion within the tumor microenvironment is mainly originated from the cells surrounding these tumors, particularly cancer-associated fibroblasts (CAFs) or tumor-associated fibroblasts (TAFs) [39]. These cells have a specially activated phenotype, marked by the expression of fibroblast activation protein-α (FAP-α) and α-smooth muscle actin (α-SMA), although with extremely heterogeneous subpopulations that may have different roles during tumor development and progression [40,41]. The increase in expression of these CAF proteins are induced by growth factors and microRNAs (miRNAs) secreted by cancer cells [22,42,43]. Once in this state, CAFs can modulate the tumor progression in several ways, such as via secreted factors, activating pro-inflammatory pathways, disrupting immune surveillance, and also by altering the ECM protein structure and stiffness [22,44,45,46,47,48]. Regarding EC, a recent study demonstrated that cells from tumor microenvironment synthesize increased ECM proteins, and the upregulation of these proteins is associated with patients’ poor prognosis and chemoresistance [38]. Moreover, using cell-derived 3D ECM, the authors showed that differences in ECM composition and structure are both crucial to EC cell response to chemotherapeutic drugs [38]. Thus, targeting ECM proteins synthesized by tumor surrounding cells could be an effective method to control cancer development and progression mainly because increased levels of ECM proteins together with their structural organization both act as a barrier for chemotherapy efficiency. A combinatory therapy that acts not only in EC cells but also in the ECM surrounding these cells should be considered. Tumor cells also need to degrade and remodel type I collagen meshwork. This is mainly performed by matrix metalloproteinases (MMPs) secreted by tumor cells as a very important feature that allows for the invasion and dissemination of cancers. Collagen molecules are synthesized as procollagens by the cell. When procollagen is secreted extracellularly, it is cleaved, assembled, and cross-linked to form collagen [12]. Indeed, the levels of such propeptides are associated with worse prognosis of patients affected by different types of cancers, being detected in their serum [49]. Moreover, it was already demonstrated that collagen fragments could induce the expression of signaling molecules, such as vascular endothelial growth factor (VEGF) and C-X-C motif chemokine receptor 4 (CXCR4), known as activators of MMP-2 and MMP-9 [50]. Over the past few decades, the notion that tumors secrete MMPs to degrade type I collagen in ECM has been very well accepted [51]. Recent studies, however, have described how cancer cells also produce their own type I collagen molecules, nevertheless presenting a different molecular weight from that of stromal cells, particularly in the case of EC [52,53]. Cancer-derived type I collagen molecules may affect tumor microenvironment and, although the precise mechanisms need to be explored, it is already known that tumor type I collagen facilitates cancer cell adhesion by modulating ECM to contribute to tumor clone formation [52,53]. Thus, one could suggest that cancer-derived type I collagen represents a promising target to improve cancer diagnosis and treatment, pointing out the need to further elucidate their function, particularly in EC. Apart from ECM remodeling promoted by tumor cells, there is also little research trying to elucidate the effect of CAFs on EC ECM remodeling. By observing the morphology of collagen fibers using multi-photon laser scanning microscopy with the second-harmonic generation, Hanley and colleagues showed that the appearance of elongated collagen fibers within CAFs is associated with poor survival rates in EC patients [54]. Moreover, the authors also showed that the formation of this collagen linear structures are specifically regulated by a CAF type that displays a myofibroblastic phenotype (αSMA-positive), suggesting that these αSMA-positive CAFs are the ones capable of regulating collagen fiber elongation and highlighting that the combined evaluation of collagen structure and αSMA-positive CAFs may be an important feature for the prognostic assessment of EC [54]. Cells sense ECM stiffness through their transmembrane receptors that are linked to the actomyosin cytoskeleton. Thus, the mechanical properties of ECM can influence growth, survival, migration, invasion, and differentiation of cells within their tissues [12,30]. Once the development of pathologic conditions, such as cancer, are accompanied by alterations in the composition, organization, and mechanical properties of ECM, these changes contribute not only to the malignant transformation but also to tumor progression and metastasis [12,30]. Cells bind to the ECM through diverse transmembrane receptors, such as integrins, syndecans, and, particularly in the case of type I collagen molecules, to discoidin domain receptors (DDRs) [55]. Upon collagen binding, DDR receptors’ cytoplasmic tails undergo autophosphorylation, creating docking sites for several other molecules, such as phosphoinositide 3-kinase (PI3K), extracellular-signal-regulated kinase (Erk), and myosin II [56,57,58]. This myosin II, together with actin, constantly checks the mechanical properties of ECM and propagates intracellular signaling pathways through a process known as mechanotransduction. It sounds very interesting, once it has already been reported that LOX, responsible for catalyzing the cross-links between collagen molecules, is highly expressed in ESCC samples, resulting in an increase in ECM stiffness [59], which, in turn, activates signaling pathways involved with malignant phenotype acquisition [12,30]. In fact, it was observed that the activation of specific signaling pathways in tumors triggered by alterations in ECM stiffness, besides altering cell morphology and migration [60]. Therefore, these data indicate that the stiffness patterns observed in EC samples [54,61] may represent the activation of several pathways involved in EC evolution. According to this hypothesis, the increase in ECM stiffness, which is observed in a vast range of tumor types [61], also contributes to tumor cells’ sustained growth, by inducing telomerase activity and, consequently, telomere stretching [62]. Such a phenomenon was also observed along EC development [63]. In this scenario, as a consequence of telomere elongation, cells are unable to arrest along cell cycle progression, conferring a limitless replication pattern, which characterizes neoplastic cells [39]. Moreover, a previous study showed that the increase in integrin expression in epithelial cells is associated with a higher telomerase enzymatic activation [64]. Thus, once integrins represent the main class of receptors interacting with ECM, one could suggest that the activity of telomerase induced by the alterations in ECM stiffness, may be mediated, at least in part, by integrin receptors. On the other hand, it has been reported that loss of integrin subunits α6, β1, and β4 is related to ESCC patients’ poor prognosis, whereas their presence is associated with increased relapse-free survival [65]. Thus, these data could suggest that the association between ECM stiffness—through integrin activation—and telomerase activity may represent an early step along esophageal carcinogenesis, once, during EC development, integrins are precociously lost [65]. In fact, it has been reported that telomere elongation is an early event related to esophageal carcinogenesis, as its association with esophageal pre-malignant lesions, such as BE, telomere elongation, and the consequently genetic instability triggered by this event, is also observed after the exposure to ethiopathological risk factors associated with EC development [63]. Finally, because integrins represent a key element during mechanotransduction, one could say that the alterations in ECM stiffness might play a central role in the esophageal tissue transformation due to its involvement in telomere elongation. In addition, a stiffer ECM will foster these signaling pathways, whereas a compliant ECM will halt them [12]. Thus, ECM stiffening through collagen cross-linking stimulates tumor cells in generating higher intracellular cytoskeletal tension, and also stronger traction forces on ECM [12]. ECM stiffening is positively correlated with the aggressive behavior of cancer cells, such that most cells migrate faster on stiffer substrates [66]. This feature can be explained because a stiffer ECM promotes cell migration and invasion by enhancing adhesion molecule clustering, focal adhesion kinase (FAK) phosphorylation, and Rho-GTPase activation, which favor the assembly of cellular protrusions such as filopodia and lamellipodia required for efficient tumor cell migration through ECM [67]. Moreover, cellular responses to mechanical stimuli also alter gene expression. For example, the expression of integrins, MMPs, and also cytoskeletal proteins are all increased in response to stiff substrates in a sort of positive feedback that enhances tumor progression and metastasis [68,69,70]. Although most of the mechanical mechanisms described above are already partially elucidated for some cancers, very little is known about these phenomena in EC. Therefore, investigating these characteristics in EC may be an interesting research topic for the field.

### 2.2. MMPs and ECM Remodeling

ECM remodeling is a very dynamic process, represented by the balance between production and degradation of proteins comprising ECM. This event plays an essential role in the maintenance of tissue homeostasis through the regulation of a myriad of interactions that constantly occur between cells and their surrounding microenvironment [71]. Nonetheless, the proteolytic enzymes play a central role in this process, by catalyzing the reactions that culminate in alterations in the chemical structure of target proteins [72]. In this context, the MMP protein family is characterized as multi-domain zinc-dependent endopeptidases and represent one of the major class of enzymes involved in ECM remodeling [72]. The different MMP subtypes are closely related to several distinct aspects of cancer development. Specifically, it has been reported that MMPs regulate key events in tumor progression, such as cell survival and invasion, metastasis development and angiogenesis, and malignant transformation [73]. MMPs are also biologically relevant for esophageal carcinogenesis. It has been previously shown that MMPs function as potential biomarkers, and distinct MMP subtypes, including MMP-1, MMP-2, MMP-3, MMP-7, and MMP-9, have been associated with EC diagnosis and/or prognosis [74,75,76,77,78,79]. In this way, among the nearly 30 distinct MMPs subtypes, MMP-2 and MMP-9 are the most strongly associated with esophageal carcinogenesis [74]. In fact, due to the breakdown of basement membrane collagen IV, MMP-2 and MMP-9 overexpression confers a poor prognosis for EC patients and is associated with late tumor stage, as well as with local invasion and presence of metastasis [74]. Hence, because aberrant expression of MMP-2 and MMP-9 plays a key role along EC progression, different studies have shed some light on the mechanisms involved in the deregulation of MMP-2 and MMP-9 expression. Accordingly, the study published by Wang and colleagues revealed that overexpression of aurora A kinases (AURKA) in ESCC cell lines triggers a signaling pathway involving other kinases, such as p38 mitogen-activated protein kinases (p38) and protein kinase B (Akt), that culminates in MMP-2 expression and activity augmentation and, consequently, enhancement of ESCC cells’ invasion abilities [80]. The involvement of the mitogen-activated protein kinase (MAPK) pathway seems to be a central mechanism in the regulation of MMP-2 and MMP-9 expression, as it it was demonstrated that a decrease in extracellular signal-regulated kinases (Erk) 1/2 and p38 phosphorylation, promoted by the tumor suppressor distinct subgroup of the Ras family member 1 (*DIRAS1*), leads to a down regulation of both MMPs [75]. Moreover, in accordance with these data, the activation of c-Jun N-terminal kinase (JNK) by E3 ubiquitin-protein ligase RAD18 also promotes an upregulation of MMP-2 and MMP-9 expression, as well as an increment in ESCC malignant cell invasion capacity [76]. In addition, the involvement of MAPK members in MMP-2 and MMP-9 expression control is likely to be also indirectly regulated by DNA damage events, because DNA polymerase iota, which acts in translesion DNA repair, is able to increase the expression of both MMPs by inducing the activation of the JNK/activator protein 1 (AP-1) axis in ESCC cells [77]. Oppositely, the study performed by Garalla and colleagues showed that the blockage of the MAPK pathway was not able to interfere in MMP-7 expression [78], although MMP-7 aberrant levels had been previously related to EC progression [79,81]. Nonetheless, in the same study, the authors demonstrated that PI3K blockage, by using the chemical inhibitor LY294002, was able to strongly abolish MMP-7 secretion by EAC cells [78]. Additionally, the increase in MMP-7 levels could also be triggered through the modulation of other pathways, and, by using a panel of ESCC malignant cell lines, it was shown that the upregulation of AP-1 induced by activin A culminated with an increase in MMP-7 levels [82]. Taken together, these data suggest that different pathways are strictly associated with the modulation of distinct MMP subtypes’ expression along esophageal carcinogenesis. Therefore, the well-established association between DNA damage and environmental factors, which characterizes the natural history of EC, may also be involved in the early deregulation of MMP function and expression. Furthermore, it seems that not only genetic alterations are related to the imbalance of MMP levels, but also to epigenetic mechanisms, such as acetylation of histones and DNA methylation, that could indirectly regulate MMP-2 and MMP-9 expression [83,84]. Additionally, obesity figures act as a crucial ethiopathological factor for EAC development. Corroborating this statement, high leptin levels, instead of adiponectin, are detected (among other hormonal alterations) in EAC patients. In this way, it was previously shown that the activation of signal transducer and activator of transcription 3 (STAT3) by leptin signaling pathway in the malignant EAC cell line OE-33 was capable of modulating the expression of MMP-2, but not that of MMP-9, which was also regulated by leptin, nevertheless in a STAT3-independent manner [85]. Because STAT3 is a recognized p38 activator, it could be reasonable to suggest that MAPK pathway activation by leptin may not be strong enough to regulate MMP-9 expression, or, as postulated by the authors, MMP-9 expression could be regulated by other mechanisms, including MAPK activators, or even other pathways mediated by leptin signaling. However, in the same work, Beales and colleagues demonstrated that adiponectin was able to antagonize leptin effects observed in EAC malignant cells, including MMP-2 and MMP-9 downregulation, thus indicating the importance of hormonal imbalance induced by obesity during EAC genesis and progression. In accordance with these observations, it was previously reported that obesity strongly influences the expression of MMP-2 and MMP-9, due to the fact that EAC cells display a great increase in the expression of these two enzymes after its co-cultivation with adipose tissue obtained from visceral fat area [86]. On the other hand, it was shown that the tumor suppressor candidate-activating transcription factor 3 (ATF3), which is significantly downregulated in malignant tissues [87], including ESCC specimens [88], coordinates a signaling cascade involved in the formation of a protein complex composed by p53, MDM2, and MMP-2, which mediates MMP-2 degradation and attenuation of tumor progression through cellular invasion inhibition [88]. Furthermore, in the same study, it was observed that cisplatin, the main chemotherapeutic agent used in EC management, was able to revert the invasive phenotype of ESCC cells, due to the induction of ATF3 expression occurring through p53 intermediation. These results sound very interesting because cisplatin is an alkylating agent that promotes DNA damage by forming adducts [89]. Otherwise, differently from other chemotherapeutic agents, cisplatin-based treatment may exhibit the best responses in EC patients, considering the additional effects exerted by this drug on the pathway that leads to MMP-2 degradation. In fact, MMP expression and activation seems to be regulated by different proteins that would be directly associated in a process orchestrated by the biological context. BE consists in one of the major hallmarks of EAC development, with GERD being the main inductor of this condition [90]. In this context, it has been previously reported that MMP-2 is activated by GERD occurring along EAC development [91]. In this way, chronic inflammation represents one of the main biological consequences promoted by GERD, with this inflammatory condition being a major driving force along EAC carcinogenesis [92]. Furthermore, nuclear factor kappa B (NF-ĸB), a key element activated during inflammation process, has been associated with the presence of invasion and metastasis during ESCC progression, by inducing epithelial mesenchymal transition (EMT) and MMP-9 expression [93]. Further, the study published by Shin and colleagues elucidated the mechanistic pathway linking the increase in MMP-9 expression and NF-ĸB by showing that protein tyrosine kinase 7 (PTK7) is able to activate NF-ĸB by a complex circuit, which involves different types of tyrosine kinase receptors, MAPK proteins, and Src [94]. In addition, it was previously reported that the pro-inflammatory interleukin 17 (IL-17) was able to induce the invasiveness of EAC cells by upregulating MMP-2 and MMP-9, and moreover increasing reactive oxygen species’ (ROS) intracellular levels [95]. In fact, Lu and colleagues showed that high levels of oxidative stress, which characterizes the bile acid reflux environment, promotes apurinic/apyrimidinic endonuclease 1 (APE1) accumulation that, in addition to the promotion of cell survival through oxidative DNA damage repair and activation of STAT3 signaling, is also capable of binding to ADP-ribosylation factor 6 (ARF6) protein. The binding of APE1 to ARF6 leads to its stabilization and, subsequently, it introduces a recycling membrane-type 1 matrix metalloproteinase (MT1-MMP) to the cellular membrane, where the MT1-MMP enzyme will activate MMP-2 [91]. Moreover, along EAC evolution, the increase in nitric oxide levels, induced by GERD, was able to promote the invasion of high-grade dysplasia Barrett’s cells by upregulating MMP-1 [96]. Of note, MMP-1 expression deregulation has been reported as a poor prognosis marker for EC development [97,98]. Therefore, it seems that a permissive environment would arise in esophageal tissue as a response to bile acid reflux insult. Specifically, the increase in NF-ĸB activation, as well as in ROS levels, caused by chronic inflammation plays a central role in the acquisition of a more invasive phenotype, as a consequence of the aberrant expression of MMPs, particularly MMP-2 and MMP-9. In addition, the relationship between GERD and MMPs along esophageal carcinogenesis seems to represent a wide event, and it has been reported that GERD condition is associated with the prevalence of polymorphisms in *MMP-1* (*1G/2G) and *MMP-3* (*6A/5A) [99]. Thus, because it is already known that polymorphisms in *MMP-1* (*1G/2G) and *MMP-3* (*6A/5A) are related to an enhanced risk for EAC development [100], these data suggest that the association between GERD and MMP polymorphisms is an early event during EAC development. Nevertheless, this scenario seems to be more complex—the impact of *MMP* polymorphisms on EC development risk modulation depends on the polymorphism itself, as well as on the *MMP* gene affected. In this way, a meta-analysis study conducted by Peng and colleagues revealed that the distinct polymorphisms present in *MMP-7* and *MMP-9* genes were not related to increased risk for EC development, and moreover, two polymorphisms found in *MMP-2* gene were associated with a diminished susceptibility of EC development [101]. Finally, it has been shown that epidermal growth factor (EGF) pathway, an important mechanism involved in the malignant transformation of several different tumors, also plays an eminent role in EC progression [102]. In this respect, in addition to the association between greater EGF and MMP-9 expression and a more invasive phenotype observed in EC tumors [103], it is known that ESCC cell line treatment with recombinant EGF leads to MMP-9 expression enhancement [104]. Of note, the study of Okawa and colleagues reported that the crosstalk between epidermal growth factor receptor (EGFR), human telomerase reverse transcriptase (hTERT), and p53 are directly associated with invasion of stromal compartment through the activation of MMP-9, but not that of MMP-2 [105]. Therefore, instead of MAPK signaling pathway, which seems to represent a central pathway involved in the regulation of MMP-2 and MMP-9, EGF signaling pathway likely participates strictly in the regulation of MMP-9, with these mechanisms being associated with PI3K activation and p53 cooperation [105,106].

### 2.3. Glycoproteins and ECM Adhesion and Migration

The activation of key cellular events depends on the interaction between cells and ECM adhesion molecules, which consists of a central mechanism represented not only by the “adhesion process itself”, but also by the activation of several signaling cascades that trigger crucial behaviors involved in the maintenance of tissue homeostasis and cancer development [107,108]. In this way, loss of E-cadherin, which plays a central role in cellular adhesion and communication by primarily mediating cell–cell adhesion, during tumor progression is directly associated with invasiveness and metastatic potential [109]. Moreover, classic malignant behaviors associated with EC progression, such as EMT, are also linked to decreased or lacking functional E-cadherin [110]. Particularly in EC, E-cadherin has drawn attention due to its great potential role as a prognostic biomarker. A meta-analysis study suggested that decreased levels of E-cadherin-positive staining are typical of undifferentiated tumor cells, and it has been proposed as a prognostic marker for ESCC patients [111]. Additionally, it was demonstrated that downregulation of E-cadherin by EC cells was directly correlated with increased risk of lymph node metastasis and advanced clinical stage [111]. Although molecular mechanisms are unclear, some target genes have been investigated and linked to reduction of E-cadherin and ESCC progression, such as p21 and cyclooxygenase-2 (COX-2) [112]. In this regard, these observations reinforce the notion that adhesion proteins, such as integrins, cadherins, and its ECM partners, play an essential role in esophageal carcinogenesis. In fact, the interaction between tumor cells and ECM mediated by adhesion glycoproteins could force the anomalous progression of cell cycle by tumor cells due to the activation of a complex circuit, which culminates in the decreased expression of the cell cycle regulators p15 and p21 [113]. Moreover, these data corroborate the previously reported association between aberrant expression of adhesion proteins, such as laminin and fibronectin, and EC progression [114,115]. Laminins are high molecular weight heterotrimeric glycoproteins produced by the association between α, β, and γ chains, playing a fundamental role for the physiology of basement membranes [116]. In fact, it has been reported that esophageal basaloid-squamous cell carcinomas displayed positive laminin and type IV collagen staining, being basement membranes thinner than in healthy esophageal tissues, and occasionally discontinuous in the EC [117]. Additionally, it has been reported that laminin receptors are progressively overexpressed in ESCC from stage I to III, such as a 67 kDa laminin receptor [114], which reinforces the association of this glycoprotein and ESCC prognosis. Nonetheless, another study demonstrated that in invasive tumors, basement membranes were partially or completely lost, depending on inflammatory pattern and epithelial organization [118]. The correlation between lack of basement membranes, inflammatory reaction in situ, and EC progression was reinforced by studies that linked these findings with secretion of proteolytic enzymes produced by cancer cells [119]. Since then, different subunits of laminins have been described and associated with the prognosis of patients affected by EC. For instance, γ2 chain of laminin-5 (Lam5 (γ2)), composed by the α3, β3, and γ2 chains, is frequently detected at elevated levels in invasive carcinomas and widely linked to recurrence in ESCC patients [120]. Indeed, co-expression of Lam5 (γ2) chain and EGFR indicates a very poor prognosis of ESCC due to high metastatic potential [121]. In fact, it seems that a mutual relationship between Lam5 (γ2) and EGF pathway exists along EC development and, besides the fact that EGF enhances Lam5 (γ2) expression [122], degradation of γ2 subunit by MT1-MMP produces a functional biological fragment, called DIII, which is able to bind and activate the EGFR, culminating with an increase in MMP-2 expression and cellular migration [123]. In accordance with these data, when in contact with MMP-1, Lam5 (γ2) cleavage enhances invasiveness and metastasis in ESCC patients [124], thus corroborating the biological consequences associated with Lam5 (γ2) degradation during ESCC progression. Moreover, laminin-5 β3 chain expression is significantly elevated in ESCC tissues in comparison with healthy esophageal tissues. This finding is directly correlated with enhanced metastatic potential and decreased life expectance in EC patients [125]. Concerning laminin-5, this protein is located in the basement membrane compartment of EC and seems to play a relevant role in ESCC carcinogenesis because it enhances tumor invasiveness potential by activating the PI3K pathway [126]. It has been demonstrated that ECM plays regulatory roles in distinct events during tumorigenesis and cancer progression; therefore, unveiling ECM composition is critical to deeper understand the molecular mechanisms through which it controls such crucial events and, consequently, to envisage potential new therapeutic strategies. Recently, Senthebane and colleagues investigated the chemotherapeutic response of ESCC cell lines cultured over 3D cell-derived ECM. In this sense, it was observed that differential expression and deposition of ECM proteins, such as collagens, fibronectin, and laminins, by ESCC cell lines was able to decrease the efficacy of different chemotherapeutic agents [38]. Further, high concentration of ECM components in the serum, including hyaluronic acid (HA) and laminin, was suggested as a potential marker for assessing upper gastrointestinal cancer development risk [127]. In EAC, high expression of laminin is associated with enhanced cell detachment, invasion, and dissemination in an osteopontin (OPN)-dependent manner [128]. Finally, the expression of laminins in EC can also be regulated by miRNAs. In this sense, it has been recently reported that laminin α1, which is widely distributed in ESCC tissues, displays an inverse correlation with the expression of miRNA-202. In other words, miRNA-202 is capable of suppressing tumor progression by targeting laminin α1 in ESCC, and high levels of laminin α1 have been associated with poor prognosis [129]. Moreover, these data are in accordance with miRNA-202 low expression observed in ESCC tissue [130,131], which was significantly associated with local invasion and metastasis [131]. Focusing on another important ECM adhesion protein, it has been already described that fibronectin is highly associated with esophageal tumorigenesis and esophageal patients’ prognosis; nevertheless, the mechanisms through which it occurs are still unclear [132]. Fibronectin is a glycoprotein present in the ECM of normal and cancerous tissues, including the esophagus, where it regulates cellular events such as adhesion, differentiation, proliferation, and metastasis. In ESCC, fibronectin is predominantly found in tumor stroma and is widely associated with lymphatic metastasis, indicating that fibronectin exerts stromal regulatory functions favoring tumor cell metastasis [133]. Inflammatory conditions also interfere with ECM organization in EC. In ESCC cells, the exposure to lipopolysaccharide (LPS) potentiated cell adhesion to fibronectin and hepatic sinusoids through toll-like receptor 4 (TLR4) signaling and selectin ligands [134]. Further, EAC-associated risk factors are capable of inducing TLR4 expression in a normal esophageal epithelium cell line, as well as in EAC cell lines (data not published). In fact, these observations sound very interesting, and the association between inflammatory process and EC development has emerged as a key event along disease development [39,135]. Furthermore, in silico analysis identified genes and signaling pathways potentially involved with EAC genesis and development, with it being detected that fibronectin 1 may represent a major target associated with metastatic capacity [136]. Indeed, fibronectin is upregulated in EAC [137], but nevertheless its contribution to cell invasion and migration is still under investigation. In this context, several authors have contributed with mechanistic proposals. One of them includes the transcriptional network of sex-determining region Y-box transcription factor 17 (SOX17) regulating fibronectin functions in ESCC tissues. Approximately 47% of ESCC patients present low levels of SOX17, which inhibits the expression of fibronectin and other genes involved with ESCC progression and metastasis [138]. On the other hand, it was demonstrated that the overexpression of fibronectin in ESCC cells could be associated with the activation of the MAPK pathway [115], as well as with miRNA regulation [139]. In this way, in ESCC patients, low miRNA-1 expression hallmarks poor clinical outcome and lymph node metastasis, whereas high expression of miRNA-1 promotes apoptosis followed by cell cycle arrest in ESCC cells, as well as fibronectin 1 organization [139]. Additionally, fibronectin may also be regulated by long noncoding RNAs (lncRNAs). It has been already reported that co-overexpression of fibronectin 1 and lncRNAs, such as lnc-ABCA12-3 (ATP Binding Cassette Subfamily A Member 12-3), in ESCC tissues is associated with tumor expansion, metastasis, and patients’ poor prognosis [140]. Furthermore, increased expression of lncRNA SPRY4-IT1 (sprouty RTK signaling antagonist 4—Intronic Transcript 1) observed in ESCC cell lines increased cell motility and favored EMT, an event strongly related to an increase in the cell motility that could be corroborated by fibronectin-positive staining [141]. In addition, during EMT-induced by hypoxia, fibronectin upregulation was directly associated with hypoxia-inducible factor 1-α (HIF-1α) secretion and downregulation of E-cadherin [142]. These data seem interesting, as hypoxia is a phenomenon largely associated with the tumor microenvironment, reinforcing the functional association of fibronectin and EMT phenotype as a tumor progression mechanism triggered by hypoxic context. Another ECM member glycoprotein involved in EC genesis and/or progression is tenascin-C. This protein controls proliferation, differentiation, and migration of different cell types in normal and tumoral tissues. In ESCC patients, this glycoprotein is predominantly detected surrounding tumor stromal cells, invading the submucosa [143]. Tenascin-C is considered as a predictive marker for EAC progression [144], and it is associated with ESCC patients’ poor prognosis due to its capacity of favoring invasion and metastasis [145,146]. In EAC, upregulation and co-overexpression of tenascin-C and fibronectin have been directly associated with poor prognosis and metastasis of the patients [137]. The mechanisms that regulate tenascin-C expression and its involvement with the development of EC remain unclear, although some efforts have already been made to shed some light on this question. Recently, Yang and colleagues demonstrated that tenascin-C displays potential in amplifying cancer stem-like properties, at least in part, indicating that tenascin-C may be involved with tumor recurrence [147]. These authors revealed that expression of tenascin-C in ESCC cells is directly associated with that of SRY-box transcription factor 2 (SOX2), a well-known cancer stem cell marker. Further, they also demonstrated that tenascin-C promotes EMT through an Akt/ Hypoxia Inducible Factor 1 Subunit Alpha (HIF1-α)-dependent manner [147]. Finally, in order to investigate ECM components with potential to regulate cellular and molecular mechanisms involved with carcinogenesis, attention has been drawn to glycoproteins that recognize membrane compartments on tumoral cells and structural ECM proteins. One of them is galectin-3, a β-galactoside-binding protein that regulates cell–cell and cell–ECM interactions and is highly associated with tumorigenesis and cancer progression [148]. In the tumor microenvironment, extracellular galectin-3 interacts with distinct ECM molecules and surface glycoproteins, such as growth factor receptors, integrins, cadherins, and members of Notch family, probably favoring the biology of cancer cells [149]. Galectin-3 interacts with galectin-3-binding protein (LGALS3BP), a receptor/ligand of galectin-3, other galectins, and ECM compounds, such as collagens and fibronectin. In ESCC cells, LGALS3BP is overexpressed in most tumors [150]. On the other hand, LGALS3BP is not considered a specific marker for ESCC progression [151]. In EC cell line Eca109, downregulation of galectin-3 induced pro-apoptotic mechanisms and inhibited proliferation, migration, and invasion [152]. Additionally, Eca109 and EC9706 cells reduced the capacity of forming tubular networks upon galectin-3 knockdown in a phenomenon associated with MMP-2 downmodulation upon galectin-3 silencing [153]. Furthermore, ESCC cell lines lacking galectin-3 expression display lower viability, mitotic index, and invasiveness capacity compared with control cells, at least in part, as a consequence of reduced EGFR endocytosis [154]. Other galectins have been linked to EC formation and progression. For instance, galectin-9 enhances antitumor immunity mediated by Tim-3^+^ dendritic cells and CD8^+^ T cells [155]. In EC cell lines, galectin-9 inhibited cell proliferation in a concentration-dependent manner and induced mitochondria-mediated apoptosis [156]. Moreover, galectin-9 showed antitumor effects in vitro, inducing apoptosis followed by high levels of caspase-cleaved cytokeratin 18, activated caspase-3, and activated caspase-9 [157]. In addition, galectin-7 overexpression in ESCC tissues has been suggested as a potential biomarker for ESCC diagnosis [158]. Furthermore, it has also been reported that some proteoglycans, such as dermatan sulfate and hyaluronan, are overexpressed in ESCC tissue [159,160]. Finally, its deregulated expression is capable of altering not only the adhesion properties of EC cells, but also of improving EC cell migration and invasion abilities through the activation of phosphorylated extracellular signal-regulated kinases (pErk1/2) and FAK [160,161].

## 3. Conclusions

**Figure 2:**
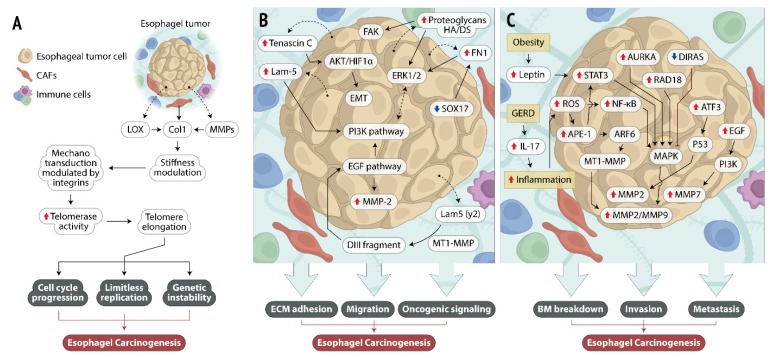
Mechanistic pathways involved in the reciprocal interactions between esophageal cancer (EC) cells and extracellular matrix (ECM). The figure illustrates the main molecules and signaling pathways altered along the interaction between tumor cells and ECM, which may represent fundamental steps for esophageal carcinogenesis. (**A**) Lysyl oxidase (LOX)- and matrix metalloproteinase (MMP)-altered expression in esophageal tumors is involved in the turnover of collagen molecules, resulting in ECM stiffness modulation that, in turn, may enhance telomerase activity, resulting in activation of signaling pathways involved with malignant phenotype acquisition, such as cell cycle progression, limitless replication, and genetic instability. (**B**) Esophageal tumor cell release increased levels of ECM adhesion molecules that activate intracellular signaling pathways involved with cell survival, enhanced cellular migration, and activation of oncogenic signaling. (**C**) Risk factors associated with esophageal tumor development (obesity and gastro-esophageal reflux disease—GERD) lead to an increase in leptin and interleukin 17 (IL-17) levels, which generates an inflammatory environment. The alterations in leptin and inflammation levels modulate the expression of several intracellular molecules that, together with other altered signaling pathways, converge to the activation of MMP expression and activity, entailing basement membrane (BM) breakdown, cellular invasion, and metastasis. Dotted arrows: molecules secreted by esophageal tumor cells; solid arrows: activation of cellular events or intracellular signaling pathways; red solid arrow: overexpressed molecules; solid blue arrow: underexpressed molecule.

ECM plays regulatory roles in the tumor microenvironment controlling tumorigenesis, progression, and metastasis. There are several biological events intrinsically linked to ECM that are crucial for cancer formation and progression, including cell differentiation and proliferation, apoptosis evasion, immunological disturbances, and inflammation. The reciprocal interactions between ECM and the surrounding EC cells may represent a fundamental step along disease development and progression, as described in Figure 2. However, the vast repertory of interactions between EC cells and ECM seems to be complex, and the mechanisms involved during the establishment of each interaction could be regulated by dozens or even hundreds of different proteins. Moreover, the singular natural history of EC, which is strongly characterized by the influence of distinct ethiopathological factors, improves the complexity of the current scenario. However, due to the scarce knowledge on the cellular and molecular mechanisms involved in this disease development, the growing evidence of ECM’s role along esophageal carcinogenesis could provide a solid base to improve EC management in the future.

## Abbreviations

α-SMA	α-smooth muscle actin
ABCA12	3 ATP Binding Cassette Subfamily A Member 12-3
Akt	protein kinase B
AP-1	activator protein 1
APE1	apurinic/apyrimidinic endonuclease 1
ARF6	ADP-ribosylation factor 6
ATF3	activating transcription factor 3
AURKA	Aurora A kinase
BE	Barrett’s esophagus
CAF	cancer-associated fibroblasts
COX-2	cyclooxygenase-2
CXCR4	C-X-C motif chemokine receptor 4
DIRAS1	Distinct Subgroup of The Ras Family Member 1
DDR	discoidin domain receptor
EAC	esophageal adenocarcinoma
EC	esophageal cancer
ECM	extracellular matrix
EGF	epidermal growth factor
EGFR	epidermal growth factor receptor
EMT	epithelial mesenchymal transition
Erk	extracellular-signal regulated kinase
ESCC	esophageal squamous cell carcinoma
FAK	focal adhesion kinase
FAP-α	fibroblast activation protein α
GERD	gastroesophageal reflux disease
HA	hyaluronic acid
HIF-1α	hypoxia-inducible factor 1-α
hTERT	human telomerase reverse transcriptase
IL-17	interleukin 17
JNK	c-Jun N-terminal kinase
Lam5 (γ2)	chain γ2 of laminin-5
LGALS3BP	galectin-3 binding protein
lncRNA	long noncoding RNA
LOX	lysyl oxidase
LPS	lipopolysaccharide
MAPK	mitogen-activated protein kinase
miRNA	microRNA
MMP	matrix metalloproteinase
MT1-MMP	membrane-type 1 matrix metalloproteinase
NF-ĸB	nuclear factor kappa B
OPN	osteopontin
P38	p38 mitogen-activated protein kinases
pErk	phosphorylated extracellular signal-regulated kinases
PI3K	phosphoinositide 3-kinase
PTK7	protein tyrosine kinase 7
ROS	reactive oxygen species
SOX	sex-determining region Y-box-box transcription factor
SPRY4-IT1	sprouty RTK signaling antagonist 4—Intronic Transcript 1
STAT3	signal transducer and activator of transcription 3
TAF	tumor associated fibroblasts
TLR4	toll-like receptor 4
VEGF	vascular endothelial growth factor
